# The Lipschitz-Volume Rigidity Problem for Metric Manifolds

**DOI:** 10.1007/s12220-026-02643-2

**Published:** 2026-10-15

**Authors:** Denis Marti

**Affiliations:** Department of Mathematics, University of Fribourg, Chemin du Musée 23, 1700 Fribourg, Switzerland

**Keywords:** Lipschitz-volume rigidity, Metric manifolds, Integral currents, Primary 53C23 Secondary 49Q15, 28A75

## Abstract

We prove a Lipschitz-volume rigidity result for 1-Lipschitz maps of non-zero degree between metric manifolds (metric spaces homeomorphic to a closed oriented manifold) and Riemannian manifolds. The proof is based on degree theory and recent developments of Lipschitz-volume rigidity for integral currents.

## Introduction

In the study of Riemannian manifolds, Lipschitz-volume rigidity has long been considered a folklore result. In its most fundamental form, the result can be stated as follows.

### Theorem 1.1

Let f:X→M be a 1-Lipschitz map of non-zero degree between two closed, oriented, connected Riemannian *n*-manifolds. If Vol(X)=Vol(M), then *f* is an isometric homeomorphism.

For a detailed proof and applications; see [7, 8]. This result has led to new developments and research in various areas of mathematics. For example, in Riemannian geometry the assumption on the volume has been replaced by a condition on the scalar curvature, leading to the celebrated Lipschitz rigidity result for spin manifolds of Llarull [19]; see also [9, 14]. More recently, there has been a growing interest in proving Lipschitz-volume rigidity type results in the field of analysis on metric spaces and geometric measure theory [2, 3, 10, 21, 25].

The proof of Theorem 1.1 relies on measure theoretic arguments and the differentiability of Lipschitz maps. Naively, this approach gives hope, that one could easily extend the result to manifolds with less differential structure. However, a simple counterexample shows that the result can fail even if *M* is a geodesic Lipschitz manifold [25]. In the present work, we are interested in weakening the conditions on the domain manifold as much as possible. We prove the following result.

### Theorem 1.2

Let n≥3 and *M* be a closed, oriented, connected Riemannian *n*-manifold. Let *X* be a metric space homeomorphic to a closed, oriented, connected smooth *n*-manifold. Suppose that $\mathcal {H}^n(X) = \mathcal {H}^n(M)$ and porous sets in *X* have measure zero. Then, every 1-Lipschitz map of non-zero degree from *X* to *M* is an isometric homeomorphism.

We say that a Borel set A⊂X is *porous* if for each x∈A there exist ε>0 and a sequence $x_n \rightarrow x$ satisfying

$$\begin{aligned} d(x_n,A) \ge \varepsilon d(x_n,x) \end{aligned}$$

for every $n \in \mathbb {N}$. By the Lebesgue density theorem, every porous set in a Riemannian manifold has measure zero. More generally, if the Hausdorff *n*-measure in *X* is doubling or *X* is a Lipschitz differentiability space, then porous sets in *X* have measure zero [6]. Moreover, if there exists c>0 such that $\mathcal {H}^n(B(x,r)) \ge cr^n$ for all x∈X and every $r \in (0,\operatorname {diam}X)$, then every porous set in *X* is negligible; see Lemma 2.4.

Theorem 1.2 also holds under weaker assumptions on *X*. Indeed, it suffices to assume that *M* is a Lipschitz manifold and an essential length space; see [25] for the definition. We do not know whether the condition on porous sets in *X* is really necessary. Note that all manifolds considered in this work are assumed to be connected and without boundary.

The author, in collaboration with Basso and Wenger, already established some Lipschitz-volume rigidity type results for metric manifolds [3, Theorem 1.4]. In fact, for surfaces, Theorem 1.2 holds without the assumption on porous sets. This has been proved in [3]. Another proof using a different approach can be found in [21]. However, both proofs of Theorem 1.2 for surfaces rely on a recent uniformization theorem for metric surfaces [22, 23]. In the case where n≥3, no such uniformization results are available and a different approach is required. In this context, it is known from [3] that the conclusion of Theorem 1.2 holds if *X* is linearly locally contractible. Notice that the theorem presented here is more general. Indeed, by [3, Theorem 4.1], linear local contractibility implies, that there exists c>0 such that $\mathcal {H}^n(B(x,r)) \ge cr^n$ for all x∈X and every $r \in (0,\operatorname {diam}X)$.

We now explain the main steps in the proof of Theorem 1.2. It is well-known that *f* is mass-preserving in this context, that is, $\mathcal {H}^n(f(B)) = \mathcal {H}^n(B)$ for every Borel B⊂X. In the proof of Theorem 1.1, one compares the volume of balls in *X* and the volume of their images in *M*. If *X* and *M* are Riemannian, we have a uniform control on the volume of sufficiently small balls in both spaces simultaneously. Since *f* is mass-preserving and 1-Lipschitz, this implies that the image of two disjoint balls cannot overlap significantly. It follows that *f* is a homeomorphism and locally bi-Lipschitz. In the situation of Theorem 1.2, we first use a result of Bate, see Theorem 2.3 below, to prove that *X* is *n*-rectifiable. Therefore, in contrast to the proof of Theorem 1.1, we obtain a uniform control of the measure of balls only after a Borel decomposition of *X*. This then implies that *f* is bi-Lipschitz only on each part of this decomposition. However, this does not suffice to conclude that *f* is an isometry. To overcome this issue, we use the theory of integral currents in the sense of Ambrosio-Kirchheim [1]. The condition on porous sets guarantees that degree theory can be applied. This allows us to use a refined version of the construction in [3] and to obtain an integral cycle in *X*. Finally, the statement then follows from the recent work of Züst [25] on Lipschitz-volume rigidity for integral currents. Such an integral current should be interpreted as an analytic analog of the fundamental class of the space. It is sometimes called a metric fundamental class and is of independent interest. We refer to [3, 20] for applications beyond Lipschitz-volume rigidity.

## Preliminaries

Let (*X*, *d*) be a metric space. We write $B(x,r)=\{y \in X :d(x,y)< r\}$ for the open ball with center x∈X and radius r>0. For U⊂X and r>0, we denote by $N_r(U) = \left\{ y \in X :d(y,U) < r\right\}$ the open *r*-neighborhood of *U*. A map f:X→Y between metric spaces is called *L*-*Lipschitz* if

$$\begin{aligned} d(f(x),f(y)) \le L d(x,y) \end{aligned}$$

for all x,y∈X. We write $\operatorname {Lip}(f)$ for the smallest constant satisfying this inequality. If *f* is injective and also its inverse is *L*-Lipschitz we say *f* is *L*-*bi-Lipschitz*.

### Degree and Orientation

We record some basic facts about the degree we use later and refer to [11] for a more detailed exposition of the topic. Let f:X→M be a continuous map between two oriented (not necessarily compact) topological *n*-manifolds *X* and *M*. Recall that throughout this article all manifolds are assumed to be connected and without boundary. Given x∈X, the relative singular homology groups $H_n(U,U\setminus \{x\})$ and $H_n(V,V\setminus \{f(x)\})$ are infinite cyclic for any open neighborhood *U* and *V* of *x* and *f*(*x*), respectively. If f(U)⊂V and f(y)≠f(x) for every $y \in U\setminus \{x\}$, then $f_* :H_n(U,U\setminus \{x\}) \rightarrow H_n(V,V\setminus \{f(x)\})$ is a well-defined homomorphism. It sends the generator of $H_n(U,U\setminus \{x\})$ to an integer multiple of the generator of $H_n(V,V\setminus \{f(x)\})$. This integer is called the *local degree* of *f* at *x* and is denoted by deg(f,x). The local degree does not depend on the choice of neighborhoods *U* and *V*. Now, let g:X→M be another continuous map with f(x)=g(x) and let U⊂X be an open neighborhood of *x*. If there exists a homotopy *H* between *f* and *g* satisfying H(y,t)≠f(x) for all $y \in U \setminus \{x\}$ and every *t*, then $\deg (f,x) = \deg (g,x)$. Moreover, the local degree satisfies the following multiplicity property; see [11, Chap. 8, Corollary 4.6]. Suppose that h:M→N is a continuous map into an oriented topological *n*-manifold *N*. If the local degrees deg(f,x) and deg(h,f(x)) are well-defined, then

$$\begin{aligned} \deg (h\circ f, x) = \deg (h,f(x)) \cdot \deg (f,x). \end{aligned}$$

In case *X* is a closed manifold, then the degree deg(f) of *f* is well-defined. As the local degree, the degree is also a homotopy invariant. More precisely, if g:X→M is another continuous map that is homotopic to *f*, then $\deg (f)=\deg (g)$. It follows [11, Chap. 8, Proposition 4.7] that for every y∈M for which the preimage $f^{-1}(y)$ is finite the local degree of *f* at each $x \in f^{-1}(y)$ is well-defined and we have

$$\begin{aligned} \deg (f) = \sum _{x \in f^{-1}(y)} \deg (f,x). \end{aligned}$$

We conclude with the following easy consequence of the homotopy invariance of the local degree. Let *M* be an oriented smooth *n*-manifold and $f:\mathbb {R}^n \rightarrow M$ be continuous. If *f* is differentiable at $x \in \mathbb {R}^n$ with non-degenerate differential $D_x f$, then $\deg (f,x)=\operatorname {sgn}(\det (D_x f))$.

### Rectifiability and Porosity

Let *X* be a complete metric space. We denote by $\mathcal {H}^n$ the Hausdorff *n*-measure on *X* and normalize it such that $\mathcal {H}^n$ equals the Lebesgue measure $\mathcal {L}^n$ on *n*-dimensional Euclidean space. A $\mathcal {H}^n$-measurable set E⊂X is said to be *n*-*rectifiable* if there exist a countable number of Borel sets $K_i \subset \mathbb {R}^n$ and Lipschitz maps $\varphi _i :K_i \rightarrow X$ such that

1 $$\begin{aligned} \mathcal {H}^n\left( E \setminus \bigcup _i \varphi _i(K_i)\right) = 0. \end{aligned}$$

The following fundamental result about rectifiable sets in metric spaces goes back to Kirchheim; see [5, Lemma 7.2] for the version presented here.

#### Lemma 2.1

Let $K \subset \mathbb {R}^n$ be a Borel set and $\varphi :K \rightarrow X$ be a Lipschitz map. Then, there exist countably many Borel sets $K_i \subset K$ such that

1. $$\mathcal {H}^n(\varphi (K) \setminus \bigcup _i \varphi (K_i)) = 0$$;
2. φ is bi-Lipschitz on each $K_i$.

It follows that for any *n*-rectifiable set E⊂X, there exists a countable collection of bi-Lipschitz maps $\varphi _i :K_i \subset \mathbb {R}^n \rightarrow X$ satisfying (1) and such that the $\varphi _i(K_i)$ are pairwise disjoint; compare to [1, Lemma 4.1]. We call such a collection $\big \{\varphi _i,K_i\big \}$ an *atlas* of *E*. Furthermore, we say that a $\mathcal {H}^n$-measurable set P⊂X is *purely* *n*-*unrectifiable* if $\mathcal {H}^n(P \cap E)=0$ for every *n*-rectifiable set E⊂X.

#### Lemma 2.2

Let *X* be a metric space with finite Hausdorff *n*-measure. Then, there exists a decomposition X=E∪P, where *E* is *n*-rectifiable and *P* is purely *n*-unrectifiable.

For the convenience of the reader we include a proof of this standard fact.

#### Proof

Put

$$\begin{aligned} s = \sup \big \{ \mathcal {H}^n(A) :A \subset X is n -rectifiable \big \}. \end{aligned}$$

For $$i \in \mathbb {N}$$, let $$E_i \subset X$$ be *n*-rectifiable such that $$s-\frac{1}{i}< \mathcal {H}^n(E_i)$$. We define $$E = \bigcup _{i \in \mathbb {N}} E_i$$. A countable union of *n*-rectifiable sets is itself *n*-rectifiable and hence, *E* is *n*-rectifiable. Let P=X\E and let B⊂X be *n*-rectifiable. Set $B^{\prime }=P\cap B$. The definition of *s* implies

$$\begin{aligned} s- \frac{1}{i} < \mathcal {H}^n(E_i) + \mathcal {H}^n(B') = \mathcal {H}^n(E_i \cup B' ) \le s \end{aligned}$$

for all $i \in \mathbb {N}$. We conclude that $\mathcal {H}^n(P \cap B) = 0$ and in particular, *P* is purely *n*-unrectifiable. □

We need a deep result due to Bate. The Euclidean norm is denoted by $\Vert \cdot \Vert$.

#### Theorem 2.3

Let *X* be a complete metric space with finite Hausdorff *n*-measure and $f:X \rightarrow \mathbb {R}^N$ be Lipschitz. Let P⊂X be purely *n*-unrectifiable. Then, for any ε>0 there exists a $(\operatorname {Lip}(f)+\varepsilon )$-Lipschitz map $g :X \rightarrow \mathbb {R}^N$ satisfying

1. $\Vert f(x)-g(x)\Vert \le \varepsilon$ for each x∈X and f(x)=g(x) whenever $d(x,P)\ge \varepsilon$;
2. $$\mathcal {H}^n(g(P)) \le \varepsilon .$$

#### Proof

The theorem is a particular instance of [5, Theorem 4.9]. More precisely, it follows from [4, Theorem 1.5] that there exists a set N⊂X with $\mathcal {H}^n(N) = 0$ such that P\N has an n-1 dimensional weak tangent field with respect to *f*. Therefore, [5, Lemma 2.8] implies that *P* is contained in the set $\tilde{A}(f,n-1)$ appearing in the statement of [5, Theorem 4.9]. Moreover, by [5, Observation 4.2] the constant $\tilde{K}(\mathbb {R}^N,n-1)$ in [5, Theorem 4.9] is equal to 1. This gives the statement of Theorem 2.3. □

We conclude the section with the following lemma.

#### Lemma 2.4

Let *X* be a compact metric space with finite Hausdorff *n*-measure. Suppose that there exists c>0 such that $\mathcal {H}^n(B(x,r)) \ge c r^n$ for each x∈X and every $r\in (0,\operatorname {diam}X)$. Then porous sets in *X* have $\mathcal {H}^n$-measure zero.

#### Proof

Let A⊂X be a porous set. Fix x∈A for the moment. Then there exists ε>0 and a sequence $x_i \rightarrow x$ satisfying

$$\begin{aligned} d(x_i,A) \ge \varepsilon d(x_i,x) \end{aligned}$$

for every $i \in \mathbb {N}$. For $i \in \mathbb {N}$, set $R_i = d(x_i,x)$ and $r_i = \frac{\varepsilon }{2}d(x_i,x)$. It follows that $B(x_i,r_i) \subset B(x,2R_i)\setminus A$ for every $i \in \mathbb {N}$. Therefore,

$$\begin{aligned} \liminf _{i\rightarrow \infty }\frac{\mathcal {H}^n(B(x,2R_i)\setminus A)}{ 2^nR_i^n} \ge \liminf _{i\rightarrow \infty }\frac{\mathcal {H}^n(B(x_i,r_i))}{2^nR_i^n}\ge \liminf _{i\rightarrow \infty }\frac{c r_i^n}{2^nR_i^n} = \frac{\varepsilon ^n}{4^n} c>0. \end{aligned}$$

Recall that a porous set is a Borel set. Hence, [12, 2.10.19 (4)] implies that

$$\begin{aligned} \lim _{r\rightarrow 0}\frac{\mathcal {H}^n(B(x,r){\setminus } A)}{\omega _n r^n}= 0 \end{aligned}$$

for almost all x∈A, where $\omega _n$ denotes the Lebesgue *n*-measure of the unit ball in $\mathbb {R}^n$. We conclude that *A* has measure zero. □

### Metric Currents

Let *X* be a complete metric space. We use the theory of metric currents developed by Ambrosio and Kirchheim [1]; see also [18]. For k≥0, we denote by $\mathcal {D}^k(X)$ the set of all (k+1)-tuples $(f,\pi _1,\dots ,\pi _k)$ of Lipschitz functions $f:X\rightarrow \mathbb {R}$ and $\pi _i:X\rightarrow \mathbb {R}$, for i=1,⋯,k, with f bounded. A metric *k*-current $T \in {{\,\mathrm{\textbf{M}}\,}}_k(X)$ is a multilinear functional $T:\mathcal {D}^k(X) \rightarrow \mathbb {R}$ that satisfies three properties that are usually referred to as the continuity property, the locality property and the finite mass property. The last property guarantees the existence of a finite Borel measure $\Vert T\Vert$ associated with *T*, called the mass measure of *T*. The *support* of T is defined as

$$ \operatorname {spt}T=\big \{ x\in X: \Vert T\Vert (B(x, r))>0 \text { for all } r>0\big \}. $$

Let $\varphi :X \rightarrow Y$ be a Lipschitz map and $T \in {{\,\mathrm{\textbf{M}}\,}}_k(X)$. The *pushforward* of *T* under φ is the metric *k*-current in *Y* defined as

$$\begin{aligned} \varphi _\# T(f,\pi _1,\dots ,\pi _k) = T(f\circ \varphi ,\pi _1\circ \varphi ,\dots ,\pi _k\circ \varphi ) \end{aligned}$$

for all $(f,\pi _1,\dots , \pi _{k})\in \mathcal {D}^k(Y)$. An important example of a *k*-current is given by the integration with respect to some $\theta \in L^1(\mathbb {R}^k)$. More specifically, each $\theta \in L^1(\mathbb {R}^k)$ induces a *k*-current $\llbracket \theta \rrbracket \in {{\,\mathrm{\textbf{M}}\,}}_k(\mathbb {R}^k)$ defined as follows

$$\begin{aligned} \llbracket \theta \rrbracket (f,\pi _1,\dots ,\pi _k) = \int _{\mathbb {R}^k} \theta f \det (D \pi ) \; d\mathcal {L}^k \end{aligned}$$

for all $(f,\pi ) = (f,\pi _1,\dots ,\pi _k) \in \mathcal {D}^k(\mathbb {R}^k).$ Motivated by the definition of a rectifiable set in a metric space, a *k*-current $T \in {{\,\mathrm{\textbf{M}}\,}}_k(X)$ is said to be *integer rectifiable* if the following holds. There exist countably many compact sets $K_i \subset \mathbb {R}^k$, and $\theta _i \in L^1(\mathbb {R}^k,\mathbb {Z})$ with $\operatorname {spt}\theta _i \subset K_i$ and bi-Lipschitz maps $\varphi _i :K_i \rightarrow X$ such that

$$ T=\sum _{i\in \mathbb {N}} \varphi _{i\#}\llbracket \theta _i\rrbracket \quad \text { and } \quad {{\,\mathrm{\textbf{M}}\,}}(T)=\sum _{i\in \mathbb {N}} {{\,\mathrm{\textbf{M}}\,}}(\varphi _{i\#}\llbracket \theta _i\rrbracket ). $$

Any such collection $\{\varphi _i,K_i,\theta _i\}$ is called a *parametrization* of *T*.

Given a *k*-current *T*, the *boundary* of *T* is defined by

$$\begin{aligned} \partial T (f,\pi _1,\dots ,\pi _{k-1}) = T(1,f,\pi _1,\dots ,\pi _{k-1}) \end{aligned}$$

for all $(f,\pi _1,\dots , \pi _{k-1})\in \mathcal {D}^{k-1}(X)$. We say an integer rectifiable *k*-current is an integral current if ∂T is a metric (k-1)-current as well. The space of all integral *k*-currents is denoted by ${{\,\mathrm{\textbf{I}}\,}}_k(X)$. Finally, an integral current without boundary is called an integral cycle.

Let *M* be a closed, oriented Riemannian *n*-manifold. There exists an integral *n*-cycle $\llbracket M\rrbracket$ naturally associated to *M*. This cycle is called the fundamental class of *M* and is defined as follows

$$ \llbracket M\rrbracket (f,\pi ) = \int _Mf\det (D\pi )\,d\hspace{-0.14em}\mathcal {H}^n $$

for all $(f, \pi )\in \mathcal {D}^n(M)$. It is a direct consequence of Stokes theorem, that this defines an integral current without boundary. We conclude this section with the recent Lipschitz-volume rigidity result of Züst for integral currents. We state a simpler version more suitable to our context.

#### Theorem 2.5

[25, Theorem 1.2] Let *M* be a closed, oriented Riemannian *n*-manifold. Let *X* be a complete metric space and $T \in {{\,\mathrm{\textbf{I}}\,}}_n(X)$ an integral cycle with ${{\,\mathrm{\textbf{M}}\,}}^\textrm{b}(T) \le \mathcal {H}^n(M)$. Then, every 1-Lipschitz map $f:\operatorname {spt}T \rightarrow M$ satisfying $f_\# T = \llbracket M\rrbracket$ is an isometry.

Given an integer rectifiable current *T* and a parametrization $\{\varphi _i,K_i,\theta _i\}$, we define the Hausdorff or Busemann mass ${{\,\mathrm{\textbf{M}}\,}}^\textrm{b}(T)$ of *T* by

$$ {{\,\mathrm{\textbf{M}}\,}}^\textrm{b}(T) =\sum _{i\in \mathbb {N}}\int _{K_i}|\theta _i| \,\textbf{J}({{\,\mathrm{\operatorname {md}}\,}}\varphi _i)\,d\mathscr {L}^n. $$

here, ${{\,\mathrm{\operatorname {md}}\,}}\varphi _i$ denotes the metric differential of $\varphi _i$. It follows from the area formula for Lipschitz maps [17] that an integer rectifiable current *T* and its Hausdorff or Busemann mass do not depend on the chosen parametrization. We refer to [25, 26] for more information.

## Proof of the Main Theorem

Throughout this section, let *M* be a closed, oriented Riemannian *n*-manifold and *X* a metric space homeomorphic to a closed, oriented smooth *n*-manifold. Suppose that $\mathcal {H}^n(X)=\mathcal {H}^n(M)$ and that there exists a 1-Lipschitz map f:X→M of non-zero degree.

We first establish some properties of *X* and *f* that hold without the additional assumption that porous sets in *X* have measure zero. The first lemma states that *f* is mass-preserving and almost injective in the measure-theoretic sense. This is a standard fact. Nevertheless, we give the simple proof because we use it frequently.

### Lemma 3.1

For every Borel set B⊂X we have $\mathcal {H}^n(f(B)) = \mathcal {H}^n(B)$. Moreover, for almost all y∈M the preimage $f^{-1}(y)$ consists of a single point.

### Proof

The map *f* has non-zero degree and is therefore surjective. This, together with the fact that *f* is 1-Lipschitz, implies that *f* is mass-preserving. Indeed, for B⊂X Borel we have

$$\begin{aligned} \mathcal {H}^n(M) = \mathcal {H}^n(f(X)) \le \mathcal {H}^n(f(X{\setminus } B)) +\mathcal {H}^n(f(B))\le \mathcal {H}^n(X{\setminus } B)+\mathcal {H}^n(B) = \mathcal {H}^n(X). \end{aligned}$$

Since $\mathcal {H}^n(X)=\mathcal {H}^n(M)$, each inequality becomes an equality. Therefore, for every Borel set B⊂X we have $\mathcal {H}^n(f(B)) = \mathcal {H}^n(B)$. Furthermore, it follows from the coarea inequality [12, 2.10.25] that

$$\begin{aligned} \mathcal {H}^n(B) = \mathcal {H}^n(f(B)) \le \int _M \mathcal {H}^0(f^{-1}(y) \cap B) \; d\mathcal {H}^n(y) \le \mathcal {H}^n(B) \end{aligned}$$

for every Borel B⊂X. We conclude that for almost all y∈M the preimage $f^{-1}(y)$ consists of a single point. □

Next, we show that *X* is *n*-rectifiable by proving that every purely *n*-unrectifiable subset P⊂X has measure zero. Since *f* is mass-preserving, it is sufficient to show that *f*(*P*) has measure zero. We outline the ideas to achieve this. First, we embed *M* into Euclidean space via a bi-Lipschitz map ι. Then, using Bate’s result (Theorem 2.3) together with a Lipschitz retraction of *M*, we perturb $g =\iota \circ f$ in a small neighborhood of *P*. This gives a surjective Lipschitz map *h* from *X* to *M* such that *h*(*P*) has small measure. Because *g* is almost injective and *h* is close to *g*, the measures of *g*(*P*) and *h*(*P*) are comparable. Since *h*(*P*) can be made arbitrarily small and *h* can be made arbitrarily close to *g*, it follows that *g*(*P*) and in particular, *f*(*P*) have measure zero.

### Lemma 3.2

The space *X* is *n*-rectifiable.

### Proof

The Riemannian manifold *M* is closed. Therefore, there exist a bi-Lipschitz embedding  and a Lipschitz retraction $$\pi :N_\delta (\iota (M)) \rightarrow \iota (M)$$ for some N≥n and δ>0. See e.g. [15, Theorem 3.10] and [16, Theorem 3.1]. Set $$g= \iota \circ f$$. Lemma 2.2 implies that there exists a decomposition X=E∪P, where *E* is *n*-rectifiable and *P* is purely *n*-unrectifiable. We have to show that $$\mathcal {H}^n(P)=0$$. By Lemma 3.1 the preimage $$g^{-1}(y) = (\iota \circ f)^{-1}(y)$$ consists of a single point for almost all $$y \in \iota (M)$$. Removing a set of measure zero from *P* if necessary, we may suppose that $$g^{-1}(g(P)) = P$$. It follows from [12, Theorem 2.2.2(1) and 2.2.3] that for every ε>0 there exists $P^{\prime }\subset P$ closed with $$\mathcal {H}^n(P \setminus P') < \varepsilon $$. In particular, it suffices to show that each closed subset $P^{\prime }\subset P$ has $$\mathcal {H}^n$$-measure zero in order to show that *P* has $$\mathcal {H}^n$$-measure zero. Hence, we may further suppose that *P* is closed. Let $$0<\varepsilon <\delta $$. We apply Theorem 2.3 to *g* and obtain a $$2\operatorname {Lip}(g)$$-Lipschitz map $$\tilde{g}:X \rightarrow \mathbb {R}^N$$ satisfying

1. $\Vert g(x)-\tilde{g}(x)\Vert \le \varepsilon '$ for each x∈X and $\tilde{g}(x)=g(x)$ whenever $d(x,P)\ge \varepsilon '$;
2. $$\mathcal {H}^n(\tilde{g}(P)) \le \varepsilon '$$.

Here, $\varepsilon^{\prime }>0$ is such that $$\varepsilon ' \operatorname {Lip}(\pi )^n < \varepsilon $$. Since π is a retraction we have $$\operatorname {Lip}(\pi ) \ge 1$$ and thus $$\varepsilon ' <\varepsilon $$. It follows that $$h = \pi \circ \tilde{g}$$ is well-defined, $$(2 \operatorname {Lip}(g) \operatorname {Lip}(\pi ))$$-Lipschitz and satisfies

(i) $\Vert g(x)-h(x)\Vert \le \varepsilon$ for each x∈X and h(x)=g(x) whenever $d(x,P)\ge \varepsilon$;
(ii) $$\mathcal {H}^n(h(P)) \le \varepsilon $$.

Notice that (1) implies (i) because $(\pi \circ g)(x) = g(x)$ for every x∈X. Moreover, the straight-line homotopy *H* between *g* and *h* satisfies $H(x,t) \in N_\delta (\iota (M))$ for every x∈X and every t∈[0,1]. Therefore, the composition of *H* and π is well-defined and *g* and *h* are homotopic as maps from *X* to ι(M). We conclude that *h* is surjective.

Recall that $g^{-1}(g(P)) = P$. Hence, the second part of (i) implies that $h^{-1}(g(P)) \setminus N_\varepsilon (P)= \emptyset$ and in particular, $h^{-1}(g(P)) \subset N_\varepsilon (P)$. It follows that $h(h^{-1}(g(P))) = g(P) \subset h(N_\varepsilon (P))$ because *h* is surjective. Thus,

$$\begin{aligned} \mathcal {H}^n(g(P)) \le \mathcal {H}^n(h(N_\varepsilon (P))) \le \varepsilon + \operatorname {Lip}(h)^n \cdot \mathcal {H}^n(N_\varepsilon (P) {\setminus } P). \end{aligned}$$

Furthermore, using that *f* is mass-preserving and ι is bi-Lipschitz, we get

$$\begin{aligned} \mathcal {H}^n(P) = \mathcal {H}^n(f(P)) \le \operatorname {Lip}(\iota ^{-1})^n \cdot \mathcal {H}^n(g(P)). \end{aligned}$$

Since *P* is closed we have $\mathcal {H}^n(N_\varepsilon (P) \setminus P)\rightarrow 0$ as $\varepsilon \rightarrow 0$. We conclude that $\mathcal {H}^n(P)=0$. This completes the proof. □

We need the following fundamental property of rectifiable sets in metric spaces. By Kirchheim [17, Theorem 9] the *n*-*dimensional density *

$$\begin{aligned} \lim _{r \rightarrow 0}\frac{\mathcal {H}^n(B(x,r))}{\omega _n r^n} \end{aligned}$$

exists and is equal to 1 for almost all x∈X. Here, $\omega _n$ denotes the volume of the Euclidean unit *n*-ball. It is easy to prove that replacing the open ball *B*(*x*, *r*) with a closed ball $\overline{B}(x,r)$ in the definition of the density does not affect the value of the limit.

### Lemma 3.3

There exist countably many compact sets $C_i \subset X$ with the following properties:

1. $$\mathcal {H}^n(X \setminus \bigcup _i C_i) = 0$$;
2. for every $i \in \mathbb {N}$ and all $x,y\in C_i$ we have
  $$\begin{aligned} \frac{1}{2} d(x,y) \le d(f(x),f(y)) \le d(x,y). \end{aligned}$$

Moreover, we may assume that for each $i \in \mathbb {N}$, there exists $K_i \subset \mathbb {R}^n$ compact and $\varphi _i:K_i \rightarrow X$ bi-Lipschitz with $\varphi _i(K_i)=C_i$.

### Proof

For r>0 and x∈X define

$$\begin{aligned} g_r(x) = \sup _{0<s< r}\frac{\mathcal {H}^n(B(x,s))}{\omega _n s^n} \quad and \quad h_r(x) = \inf _{0<s<r}\frac{\mathcal {H}^n(\overline{B}(x,s))}{\omega _n s^n}. \end{aligned}$$

Since *X* is *n*-rectifiable we conclude that $g_r(x), h_r(x) \rightarrow 1$ as r→0 for almost all x∈X. Moreover, the functions $g_r$ and $h_r$ are lower semicontinuous and upper semicontinuous, respectively; see [24, Chap. 1, Remark 3.2]. It follows from Egoroff’s theorem [12, 2.3.7] and the Borel regularity of $\mathcal {H}^n$ that for every ε>0, there exists a compact set C⊂X such that $\mathcal {H}^n(X \setminus C) < \varepsilon$ and both $g_r$ and $h_r$ converge uniformly on *C*. By applying this observation repeatedly to a sequence of ε>0 converging to zero we conclude the following. There exist countably many compact sets $C_i \subset X$ such that

(i) $$\mathcal {H}^n(X \setminus \bigcup _i C_i ) =0$$;
(ii) for every $i \in \mathbb {N}$ and every δ>0 there exist r>0 such that
  $$\begin{aligned} 1-\delta \le \inf _{0<s< r}\frac{\mathcal {H}^n(\overline{B}(x,s))}{\omega _n s^n} \le \sup _{0<s < r}\frac{\mathcal {H}^n(B(x,s))}{\omega _n s^n} \le 1+\delta \end{aligned}$$
  for each $$x \in C_i$$.

Fix $i \in \mathbb {N}$ for the moment. Let δ>0 be such that $4^n 5 \delta <1$. By the coarea inequality we have

$$\begin{aligned} \int _0^r \mathcal {H}^{n-1}(\{y\in X :d(x,y)=s\}) \;ds \le \mathcal {H}^n(B(x,r))<\infty , \end{aligned}$$

for every x∈X and each r>0. It follows that the $\mathcal {H}^n$-measure of the open *B*(*x*, *s*) and the closed ball $\overline{B}(x,s)$ are equal for every x∈X and almost every s>0. Therefore, (ii) implies that there exists r>0 such that

2 $$\begin{aligned} \omega _n s^n - \delta \omega _n s^n \le \mathcal {H}^n(B(x,s)) \le \omega _n s^n + \delta \omega _n s^n \end{aligned}$$

for every $x \in C_i$ and almost all s∈(0,r). Since *M* is a closed Riemannian manifold, we may assume that (2) also holds for every x∈M and each s∈(0,r). Now, let $x,y \in C_i$ with d(x,y)<2r and suppose that $d(f(x),f(y)) < \frac{1}{2}d(x,y)$. Set t=d(f(x),f(y)) and let $s \in (\frac{3}{4}t,t)$ be such that (2) holds for *B*(*x*, *s*) and *B*(*y*, *s*). By the above and using that *f* is mass-preserving, we get

$$\begin{aligned} \mathcal {H}^n(f(B(x,s)\cup B(y,s))) = \mathcal {H}^n(B(x,s) \cup B(y,s)) \ge 2\omega _n s^n - 2\delta \omega _n s^n. \end{aligned}$$

On the other hand, we have $f(B(x,s)\cup B(y,s)) \subset B(f(x),s) \cup B(f(y),s)$, because *f* is 1-Lipschitz. Furthermore, the intersection B(f(x),s)∩B(f(y),s) contains a ball of radius *s*/4. Therefore,

$$\begin{aligned} \mathcal {H}^n(f(B(x,s)\cup B(y,s))) \le \left( 2-\frac{1}{4^n}\right) \omega _ns^n + \left( 2+\frac{1}{4^n}\right) \delta \omega _n s^n. \end{aligned}$$

However, by our choice of δ we have

$$\begin{aligned} 2\omega _n s^n - 2\delta \omega _n s^n > \left( 2-\frac{1}{4^n}\right) \omega _ns^n +\left( 2+\frac{1}{4^n}\right) \delta \omega _n s^n. \end{aligned}$$

This is clearly a contradiction. We conclude that $\frac{1}{2}d(x,y) \le d(f(x),f(y))$ for all $x,y \in C_i$ with d(x,y)≤2r. The claim follows by further partitioning each $C_i$ using the *n*-rectifiability of *X*. □

The next lemma is the only place where we use the fact that porous sets in *X* have measure zero. Specifically, we use the following consequence of this fact. Let A⊂X be Borel and ε>0, then for almost all x∈A there exists r>0 such that for every y∈B(x,r) there exists z∈A satisfying $d(y,z) \le \varepsilon d(y,x)$.

### Lemma 3.4

Let $g,h :X \rightarrow \mathbb {R}^n$ be Lipschitz and C⊂X be a Borel set such that g(x)=h(x) for all x∈C. If porous sets in *X* have measure zero, then for almost all $y \in \mathbb {R}^n$ and every $x \in g^{-1}(y) \cap C$ we have $\deg (g,x) = \deg (h,x)$.

### Proof

First, we prove the statement in the special case when C=φ(K) for some compact set $K \subset \mathbb {R}^n$ and a bi-Lipschitz map $\varphi :K \rightarrow X$. By applying Lemma 2.1 to $\eta = g \circ \varphi = h \circ \varphi$ and using that φ is bi-Lipschitz we obtain countably many compact sets $C_i \subset C$ such that

- $$\mathcal {H}^n(g(C) \setminus \bigcup _i g(C_i)) = \mathcal {H}^n(h(C) \setminus \bigcup _i h(C_i)) = 0$$;
- *g* and *h* are bi-Lipschitz on each $C_i$.

For $i \in \mathbb {N}$, let $N_i\subset C_i$ be the set of points $x\in C_i$ such that $C_i$ is porous at *x*, that is, the property stated before the lemma does not hold at *x*. By our assumption $N = \bigcup _i N_i$ has measure zero and in particular, $\mathcal {H}^n(g(N)) = \mathcal {H}^n(h(N)) = 0$. Now, let $y \in \mathbb {R}^n$ be such that $g^{-1}(y) \subset \bigcup _i C_i\setminus N$ and the preimage $g^{-1}(y)$ is finite. Notice that by the above and the coarea inequality, this holds for almost all $y \in \mathbb {R}^n$. Let $x \in g^{-1}(y)$ and $i \in \mathbb {N}$ with $x \in C_i$. Then there exists L≥1 such that *g* and *h* are *L*-Lipschitz on *X* as well as *L*-bi-Lipschitz on $C_i$. Furthermore, there exists r>0 with the following property. For every z∈B(x,r) there exists $z' \in C_i$ satisfying $d(z,z')\le (3L)^{-2} d(x,z)$. For such *z* we have

3 $$\begin{aligned} \Vert g(x)-g(z)\Vert \ge \Vert g(x)-g(z')\Vert - \Vert g(z)-g(z')\Vert \ge \frac{7}{9L}d(x,z). \end{aligned}$$

Notice that the same holds if *g* is replaced by *h*. Moreover,

4 $$\begin{aligned} \Vert g(z)-h(z)\Vert \le \Vert g(z)-g(z')\Vert + \Vert h(z')-h(z)\Vert \le 2L d(z,z') \le \frac{2}{9L}d(x,z). \end{aligned}$$

It follows from (3) that for all $z \in B(x,r) \setminus \{x\}$ we have g(z)≠g(x) and h(z)≠h(x). Therefore, the local degrees deg(g,x) and deg(h,x) are well-defined. Furthermore, let $H:X\times [0,1] \rightarrow \mathbb {R}^n, (z,t) \mapsto t g(z) + (1-t) h(z)$ be the straight-line homotopy between *g* and *h*. By (4) we have

$$\begin{aligned} \Vert H(z,t)-g(z)\Vert \le \Vert (1-t) (g(z)-h(z))\Vert \le (1-t) \frac{2}{9\,L}d(x,z) \end{aligned}$$

for every z∈B(x,r) and every t∈[0,1]. Together with (3) we get

$$\begin{aligned} \Vert H(z,t)-g(x)\Vert \ge \Vert g(x)-g(z)\Vert - \Vert H(z,t)-g(z)\Vert \ge (1-t) \frac{5}{9\,L}d(x,z). \end{aligned}$$

This shows that *H* satisfies H(z,t)≠g(x) for every $z \in B(x,r)\setminus \{x\}$ and every t∈[0,1]. We conclude from the homotopy invariance of the local degree that $\deg (g,x)= \deg (h,x)$. This completes the proof in the special case. We now prove the general statement. Since *X* is *n*-rectifiable, C⊂X is *n*-rectifiable as well. Thus, there exist countably many compact sets $K_i \subset \mathbb {R}^n$ and bi-Lipschitz maps $\varphi _i :K_i \rightarrow X$ such that the $C_i = \varphi _i(K_i)$ are pairwise disjoint and $N = C \setminus \bigcup _{i \in \mathbb {N}} C_i$ has $\mathcal {H}^n$-measure zero. We have

$$\begin{aligned} g^{-1}(y) \cap C = g^{-1}(y)\cap \left( \bigcup _{i \in \mathbb {N}} C_i\right) = \bigcup _{i\in \mathbb {N}} g^{-1}(y)\cap C_i \end{aligned}$$

for almost all $y \in \mathbb {R}^n$ because g(N)=h(N) has $\mathcal {H}^n$-measure zero. Therefore, the first part of the proof implies that for almost all $y \in \mathbb {R}^n$ and every $x \in g^{-1}(y) \cap C$ the local degrees deg(g,x) and deg(h,x) coincide. This completes the proof. □

We are now in a position to construct a non-trivial integral cycle in *X*.

### Lemma 3.5

If porous sets in *X* have measure zero, then there exists an integral cycle $T \in {{\,\mathrm{\textbf{I}}\,}}_n(X)$. Moreover, the parametrization $\{\varphi _i,K_i,\theta _i\}$ of *T* is such that $\{\varphi _i,K_i\}$ is an atlas of *X* and $|\theta _i(x)|=1$ for every $i\in \mathbb {N}$ and almost all $x \in K_i$.

### Proof

Recall that *X* is *n*-rectifiable. Lemma 3.3 implies there exists an atlas $\big \{ \varphi _i,K_i\big \}$ of *X*

such that *f* is 2-bi-Lipschitz on each $C_i = \varphi _i(K_i)$. For $i \in \mathbb {N}$, set $g_i = f \circ \varphi _i$. By partitioning each $K_i$ if necessary, we can suppose that each $f(\varphi _i(K_i))$ is contained in an open ball in *M* that is bi-Lipschitz homeomorphic to an open ball in $\mathbb {R}^n$. Using McShane’s extension theorem, we conclude that, for each $i\in \mathbb {N}$, there exists a Lipschitz extension $\tilde{g}_i$ of $g_i$ to some open neighborhood $U_i$ of $K_i$. Since every $\tilde{g_i}$ is Lipschitz and bi-Lipschitz on $K_i$, it follows that for each $i \in \mathbb {N}$ and for almost all $z \in K_i$, the extension $\tilde{g}_i$ is differentiable and the differential $D_z \tilde{g}_i$ is non-degenerate. Therefore, for such *z* we have $\deg (\tilde{g_i},z) = \operatorname {sgn}(\det (D_z \tilde{g}_i))$ and in particular, the local degree $\deg (\tilde{g_i},z)$ equals 1 or -1. Define

$$\begin{aligned} T= \sum _i \varphi _{i}\#\llbracket \theta _i\rrbracket , \end{aligned}$$

where $\theta _i(z)= \deg (\tilde{g_i},z)$. One shows exactly as in the proof of [3, Lemma 5.1] that *T* is an integer rectifiable *n*-current of finite mass. It remains to prove that ∂T=0. More precisely, we have to show that

$$\begin{aligned} T(1,\pi ) = \sum _i \varphi _{i\#} \llbracket \theta _i\rrbracket (1,\pi ) =0 \end{aligned}$$

for all $\pi \in \operatorname {Lip}(X,\mathbb {R}^n)$. Fix $i \in \mathbb {N}$ and $\pi \in \operatorname {Lip}(X,\mathbb {R}^n)$ for the moment. Let $\psi :\mathbb {R}^n \rightarrow \mathbb {R}^n$ be a Lipschitz extension of $\pi \circ \varphi _i$ and $\eta :M \rightarrow \mathbb {R}^n$ be a Lipschitz extension of $\pi \circ f^{-1} :f(C_i) \rightarrow \mathbb {R}^n$. Notice that η satisfies $\eta \circ \tilde{g}_i (z) = \pi \circ \varphi _i(z)$ for all $z \in K_i$. In particular, $\eta \circ \tilde{g_i}$ is another Lipschitz extension of $\pi \circ \varphi _i$.

**Figure:**
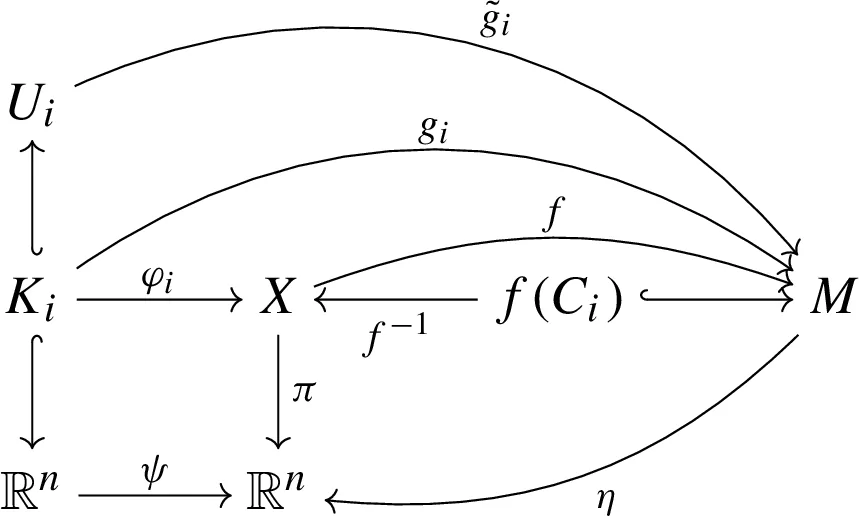

Let A⊂M be the set of points x∈M where η is not differentiable. By Rademacher’s theorem *A* has $\mathcal {H}^n$-measure zero. Using that *f* is mass-preserving and surjective we get

$$\begin{aligned} \mathcal {H}^n(f^{-1}(A)) = \mathcal {H}^n(f(f^{-1}(A))) = \mathcal {H}^n(A) = 0. \end{aligned}$$

We conclude that $\varphi _i^{-1}(f^{-1}(A))$ has $\mathcal {H}^n$-measure zero because $\varphi _i$ is bi-Lipschitz. Furthermore, it is not difficult to show that if $z\in K_i$ is a density point of $K_i$ and the derivatives $D_z(\eta \circ \tilde{g_i})$ and $D_z\psi$ exist, then they are equal. We define $B_1 \subset K_i$ to be the set of points *z* such that the following holds. The Lipschitz maps ψ, $\tilde{g_i}$ and η are differentiable at *z* and $\tilde{g}_i(z)$, respectively, as well as $D_z(\eta \circ \tilde{g_i}) = D_z\psi$. Since almost every point in $K_i$ is a density point of $K_i$ and $\mathcal {H}^n(\varphi _i^{-1}(f^{-1}(A))) = 0$, we have $\mathcal {H}^n( K_i \setminus B_1) = 0$. Moreover, let $B_2 \subset B_1$ be the set of points *z* such that the derivative $D_z\psi$ is non-degenerate. The area formula implies

$$\begin{aligned} \mathcal {H}^n(\psi (K_i{\setminus } B_2)) {\le } \int _{\mathbb {R}^n} \mathcal {H}^0((K_i{\setminus } B_2) \cap \psi ^{-1}(y)) \;\mathcal {L}^n(y){=} \int _{K_i{\setminus } B_2} \det (D_z\psi ) \;\mathcal {L}^n(z) =0. \end{aligned}$$

Finally, it follows from the coarea inequality that the preimages $\psi ^{-1}(y)\cap K_i$ and $(\eta \circ \tilde{g}_i)^{-1}(y)\cap K_i$ are finite for almost all $y\in \mathbb {R}^n$. The multiplicity of the degree, when combined with the preceding observations, implies

$$\begin{aligned} \operatorname {sgn}(\det D_z\psi )= \operatorname {sgn}(\det D_z(\eta \circ \tilde{g_i})) = \deg (\eta \circ \tilde{g_i},z) = \deg (\tilde{g_i},z) \cdot \deg (\eta ,\tilde{g_i}(z)) \end{aligned}$$

for almost all $y \in \mathbb {R}^n$ and every $z \in \psi ^{-1}(y)\cap K_i$. Therefore,

$$\begin{aligned} \varphi _{i\#}\llbracket \theta _i\rrbracket (1,\pi )&= \int _{K_i} \theta _i(z) \det (D_z \psi ) \; d\mathcal {L}^n(z) \\&= \int _{K_i} \theta _i(z) \operatorname {sgn}(\det (D_z \psi )) |\det (D_z \psi )| \; d\mathcal {L}^n(z) \\&= \int _{K_i} \deg (\eta ,\tilde{g_i}(z)) |\det (D_z \psi )| \; d\mathcal {L}^n(z). \end{aligned}$$

The area formula yields

$$\begin{aligned} \int _{K_i} \deg (\eta ,\tilde{g_i}(z)) |\det (D_z \psi )| \; d\mathcal {L}^n(z)= \int _{\mathbb {R}^n}\left( \sum _{z\in \psi ^{-1}(y) \cap K_i} \deg (\eta , \tilde{g}_i(z))\right) d\mathcal {L}^n(y). \end{aligned}$$

Recall that $C_i = \varphi _i(K_i)$ and $\psi (z) = (\pi \circ \varphi _i)(z)$ for every $z \in K_i$. Using that $\varphi _i$ is bi-Lipschitz on $K_i$ we get $z \in \psi ^{-1}(y)\cap K_i$ if and only if $\varphi _i(z) \in \pi ^{-1}(y)\cap C_i$ for every $y \in \mathbb {R}^n$. Hence,

5 $$\begin{aligned} \sum _{z\in \psi ^{-1}(y) \cap K_i} \deg (\eta , \tilde{g}_i(z)) = \sum _{\varphi _i(z) \in \pi ^{-1}(y)\cap C_i} \deg (\eta , \tilde{g}_i(\varphi _i(z))) \end{aligned}$$

for all $y \in \mathbb {R}^n$. For $z \in K_i$, we have $\tilde{g}_i(z) =(f\circ \varphi _i)(z)$ and thus, $\deg (\eta ,\tilde{g}_i(z)) = \deg (\eta ,f(x))$ for $x= \varphi _i(z)\in C_i$. Therefore,

6 $$\begin{aligned} \sum _{\varphi _i(z) \in \pi ^{-1}(y)\cap C_i} \deg (\eta , \tilde{g}_i(\varphi _i(z))) = \sum _{x\in \pi ^{-1}(y) \cap C_i} \deg (\eta , f(x)) \end{aligned}$$

for every $y \in \mathbb {R}^n$. Combining (5) and (6) we obtain

$$\begin{aligned} \int _{\mathbb {R}^n}\left( \sum _{z\in \psi ^{-1}(y) \cap K_i} \deg (\eta , \tilde{g}_i(z))\right) d\mathcal {L}^n(y) = \int _{\mathbb {R}^n}\left( \sum _{x\in \pi ^{-1}(y) \cap C_i} \deg (\eta , f(x))\right) d\mathcal {L}^n(y). \end{aligned}$$

By Lemma 3.1 the preimage $f^{-1}(z)$ consists of a single point for almost all z∈M and *f* is mass-preserving. Hence, the additivity property of the degree implies that $\deg (f,x) = \deg (f)$ for almost all x∈X. Moreover, for each $x \in C_i$ we have $(\eta \circ f)(x) = \pi (x)$. It follows from Lemma 3.4 and the multiplicity of the degree that

$$\begin{aligned} \deg (\pi ,x) = \deg (\eta \circ f,x) = \deg (\eta ,f(x))\cdot \deg (f) \end{aligned}$$

for almost all $y\in \mathbb {R}^n$ and every $x \in \pi ^{-1}(y) \cap C_i$. In particular,

$$\begin{aligned}&\int _{\mathbb {R}^n}\left( \sum _{x\in \pi ^{-1}(y) \cap C_i} \deg (\eta , f(x))\right) d\mathcal {L}^n(y)= \\&\frac{1}{\deg (f)}\int _{\mathbb {R}^n}\left( \sum _{x\in \pi ^{-1}(y) \cap C_i} \deg (\pi , x)\right) \,d\mathcal {L}^n(y). \end{aligned}$$

We conclude that

$$\begin{aligned} T(1,\pi ) = \sum _i \varphi _{i\#} \llbracket \theta \rrbracket (1,\pi ) = \frac{1}{\deg (f)} \int _{\mathbb {R}^n} \left( \sum _{x \in \pi ^{-1}(y)} \deg (\pi ,x) \right) \, d\mathcal {L}^n(y) \end{aligned}$$

for every $\pi \in \operatorname {Lip}(X,\mathbb {R}^n)$. It follows from the additivity property of the degree that for every $y \in \mathbb {R}^n$ for which the preimage $\pi ^{-1}(y)$ is finite we have

$$\begin{aligned} \sum _{x \in \pi ^{-1}(y)} \deg (\pi ,x) = \deg (\pi ). \end{aligned}$$

Since *X* is closed, we have $\deg (\pi ) = 0$, see e.g. [11, p. 269]. This shows T(1,π)=0 and completes the proof. □

Finally, we can give the proof of the main theorem.

### Proof of Theorem 1.2

Let $T \in {{\,\mathrm{\textbf{I}}\,}}_n(X)$ be the integral cycle given by Lemma 3.5 and $\{\varphi _i,K_i,\theta _i\}$ its parametrization. Recall that $|\theta _i(x)| =1$ for every $i \in \mathbb {N}$ and almost all $x \in K_i$. Therefore, by the definition of the Hausdorff or Busemann mass we have

$$ {{\,\mathrm{\textbf{M}}\,}}^\textrm{b}(T) =\sum _{i\in \mathbb {N}}\int _{K_i}|\theta _i| \,\textbf{J}({{\,\mathrm{\operatorname {md}}\,}}\varphi _i)\,d\mathscr {L}^n =\sum _{i\in \mathbb {N}}\int _{K_i} \,\textbf{J}({{\,\mathrm{\operatorname {md}}\,}}\varphi _i)\,d\mathscr {L}^n. $$

Since $\{\varphi _i,K_i\}$ is an atlas of *X*, the area formula implies ${{\,\mathrm{\textbf{M}}\,}}^\textrm{b}(T)=\mathcal {H}^n(X)$ and in particular, ${{\,\mathrm{\textbf{M}}\,}}^\textrm{b}(T)=\mathcal {H}^n(M)$. Moreover, again using that $\{\varphi _i,K_i\}$ is an atlas of *X*, we have $\operatorname {spt}T = X$. By partitioning the parametrization of *T* if necessary, and by Lemmas 3.1 and 3.3, we may assume that the following holds: *f* is bi-Lipschitz on each $C_i = \varphi _i(K_i)$ and the preimage $f^{-1}(y)$ consists of a single point for every $i \in \mathbb {N}$ and for all $y \in f(C_i)$. It follows that $\{f\circ \varphi _i,K_i,\theta _i\}$ is a parametrization of the integral cycle $f_\# T \in {{\,\mathrm{\textbf{I}}\,}}_n(M)$. Furthermore, using in addition that *f* is surjective, we conclude that $\{f\circ \varphi _i,K_i\}$ is an atlas of *M*. Thus, as before, the definition of the Hausdorff or Busemann mass ${{\,\mathrm{\textbf{M}}\,}}^\textrm{b}$ and the area formula imply that ${{\,\mathrm{\textbf{M}}\,}}^\textrm{b}(f_\#T)=\mathcal {H}^n(M) = {{\,\mathrm{\textbf{M}}\,}}^\textrm{b}(\llbracket M\rrbracket )$. Since *M* is a closed, oriented Riemannian manifold, the constancy theorem applies. That is, each integral *n*-cycle $S\in {{\,\mathrm{\textbf{I}}\,}}_n(M)$ is of the form $S = k \cdot \llbracket M\rrbracket$ for some integer $k \in \mathbb {Z}$; see [13, Corollary 3.13]. For $S = f_\#T$, we have that *k* is either 1 or -1 because ${{\,\mathrm{\textbf{M}}\,}}^\textrm{b}(f_\#T)={{\,\mathrm{\textbf{M}}\,}}^\textrm{b}(\llbracket M\rrbracket )$. Therefore, by replacing *T* by -T if necessary, we may suppose that $f_\# T = \llbracket M\rrbracket$. We conclude that *T* satisfies all conditions of Theorem and hence, f:X→M is an isometric homeomorphism. □

## Data Availability

The paper has no associated data.
